# Determinants of vaccine adherence among non-dialysis chronic kidney disease patients in Qatar

**DOI:** 10.5339/qmj.2023.33

**Published:** 2024-01-06

**Authors:** Mhd Baraa Habib, Khaled Ali, Alaa Rahhal, Ibrahim Obeidat, Mohammed Altermanini, Bisher Sawaf, Tarek Abdel latif, Abdullah Hamad, Iheb Bougmiza, Sahar Ismail Aly, Khaled Mohamed Mahmoud

**Affiliations:** ^1^Internal Medicine Department, Hamad Medical Corporation, Doha, Qatar; ^2^Community Medicine Department, Hamad Medical Corporation, Doha, Qatar Email:kali15@hamad.qa ORCID iD: 0000-0002-8073-4375; ^3^Pharmacy Department, Hamad Medical Corporation, Doha, Qatar; ^4^Nephrology Division, Department of Medicine, Hamad Medical Corporation, Doha, Qatar; ^5^Community Medicine Department, Primary Health Care Corporation, Doha, Qatar

**Keywords:** vaccine, chronic kidney disease, adherence, influenza, pneumococcal, hepatitis B

## Abstract

Introduction: Chronic kidney disease (CKD) is a global health problem. Reduced innate and adaptive immunological responses predispose CKD patients to infections. Despite the clinical and epidemiological importance of CKD and the great value of vaccination as a prophylactic measure, the utilization of recommended vaccines in Qatar has not yet been evaluated.

Methods: We conducted a cross-sectional study to estimate the level of influenza, pneumococcal, and hepatitis B vaccination and the predictors of adherence to these recommended vaccines among non-dialysis CKD patients receiving renal ambulatory care in Qatar from 1 September 2020 to 30 April 2021. Complete vaccination was defined as receiving the three vaccines, and partial vaccination was defined as receiving one or two vaccines. The full and partial vaccination predictors were assessed using multivariate logistic regression and reported as odds ratio (OR) with p<0.05 indicating statistical significance.

Results: 416 non-dialysis CKD patients were included in our analysis. 73% were males; the mean age was 56 ± 15 years. More than 50% of the patients were from the Middle East, followed by 36% from Asia. Most patients had concurrent hypertension, concurrent diabetes mellitus, and were stage V CKD. Only 12% of the patients were fully vaccinated, while 73% received partial vaccination. The predictors of vaccination included age, gender, Asian origin, employment, living conditions, concurrent medical conditions, CKD stage, allergy to medications, and use of injectable medications. Only stage V CKD positively predicted adherence to full and partial vaccinations in non-dialysis CKD patients.

Conclusion: There is very low adherence to the recommended vaccines in CKD patients, with a prevalence of complete vaccination of 12% only. Increased public awareness about the importance of vaccination in CKD may improve the adherence rates among these patients in Qatar.

## Introduction

Chronic kidney disease (CKD) is a global health problem, as the number of patients with end-stage renal disease (ESRD) in the United States has increased from approximately 10,000 in 1973 to around 700,000 in 2015.^1^ Worldwide prevalence of CKD was estimated to be 10.4% among men and 11.8% among women, with higher rates in low and middle-income countries.^2^ Moreover, the yearly incidence of CKD was estimated to be around 1700 per million, according to a cohort study of a 5.5-year follow-up period (study population base of 405,000).^3^

Decreased kidney function is established when the glomerular filtration rate is below 60 mL/min/1.73 m^2^. This state is defined as chronic kidney disease (CKD) only if it is persistent for at least three months so that it can be distinguished from Acute Kidney Injury (AKI). Urine albumin excretion of more than 30 mg/day is another criterion that indicates kidney damage and has to be persistent for at least three months too.^4^ Repeating these tests is essential to evaluate chronicity and for patients with test results near the threshold values.^5^ Staging chronic kidney disease is critical to effectively manage the case, predict and prevent possible complications, and delay the disease progression. Staging involves three main aspects: the cause of the disease, the GFR (glomerular filtration rate) stage, and the albuminuria stage.^4^

High morbidity and high demand for health care services are observed in both non-dialysis and dialysis CKD patients.^6^ The rate of hospitalization was around double among dialysis patients in comparison to early-stage CKD patients.^7^ Regarding disability-adjusted life years (DALYs), the rank of CKD increased from 30 in 1990 to 20 in 2015, according to the Global Burden of Disease report.^8^

A study from Qatar has demonstrated that age above 60 and smoking were associated with more progressive CKD.^9^ Also, it showed that having Hypertension and/or Diabetes mellitus as the leading cause of CKD was associated with a worse prognosis and more significant GFR decline.^9^

The second most important cause of death among CKD patients is infection. This is why vaccination is considered one of the essential factors in preventing adverse outcomes in these patients.^10^ The primary recommended vaccines are Hepatitis B, Pneumococcal, and Influenza.^10^ Early Hepatitis B vaccination has better seroconversion rates, so CKD patients in the early stages should receive the vaccine as soon as possible.^11^ Influenza vaccine has a massive benefit for all chronic disease patients, including CKD, and it is recommended since it reduces hospitalization rate and, hence, mortality rate.^12^

Pneumococcal vaccine is also important in CKD patients since their weakened immunity decreases their ability to defend against these organisms, making them more susceptible to severe Pneumococcal infection.^13^

Using these recommended vaccines among non-dialysis CKD patients is not well-studied.^14^ Despite the clinical and epidemiological importance of CKD and the great value of vaccination as a preventive measure, the utilization of the recommended vaccines in Qatar has not yet been evaluated. It is essential to advocate vaccination among non-dialysis CKD patients, as the number of dialysis patients in Qatar is predicted to increase by 2030.^15^ Vaccination promotion to the adult population in Qatar is done through the Ministry of Public Health, Hamad Medical Corporation, and Primary Health Care Corporation campaigns.

## Methods

### Study Design, Sampling, Data Collection

A retrospective analytical cross-sectional study was conducted at Fahad Bin Jasem Hospital, Department of Nephrology, Doha, Qatar. All necessary data were gathered from the electronic medical records. All patients who visited the clinic between September 1, 2020, and April 30, 2021, and who met the inclusion criteria were included in this study. The total number of patients inquired about in the study was 416.

### Objectives

Primary objective: To report adherence to recommended vaccinations (Hepatitis B, pneumococcal, and influenza) among non-dialysis CKD patients.

Secondary objective: To determine the predictors of receiving the recommended vaccinations (Hepatitis B, pneumococcal, and influenza vaccinations) among non-dialysis CKD patients using patient- and disease-related factors.

### Inclusion and Exclusion Criteria

We included patients older than 18 who are diagnosed with CKD (all-stage CKD patients except for patients on dialysis). We excluded patients who had had kidney transplantation, were on dialysis, or had a severe adverse or hypersensitive response to vaccines.

### Measurements and Outcomes

The computerized patient files were checked, and information was taken for two sections: (1) demographic factors, including age, gender, nationality, occupation, housing conditions, the existence of chronic illnesses other than CKD, the stage of renal disease, the presence of allergies, the number of chronic medications, and the presence of injectable chronic medications. (2) the status of Hepatitis B, pneumococcal, and influenza vaccinations.

Evaluated outcomes were the prevalence of complete vaccination, defined as receiving the three vaccines, and the majority of partial vaccination, defined as receiving one or two vaccines. The outcomes were reported as percentages, along with full and partial vaccination predictors.

### Data Analysis

Data were analyzed using a statistical package for the social sciences (SPSS) version 28.0 software (IBM Corporation Armonk, New York, USA). For descriptive statistics, we used the mean and standard deviation, or the median and interquartile range, depending on the distribution of variables. For categorical data, we utilized percentages wherever they were relevant. Percentages were provided for the prevalence of both full and partial vaccination. Multivariate logistic regression was used to assess the full and partial vaccination predictors. The results were given as odds ratios (OR), with a p-value of < 0.05 representing statistical significance. Tests were done at a 2-sided 5% level of significance. The work was done in line with the Strengthening the reporting of observational studies in epidemiology (STROBE) statement.

### Ethical Consideration

The Institutional Review Board at Hamad Medical Corporation approved the study, IRB register number MRC-01-21-530. Due to the retrospective nature of data collection and analysis, a waiver for the requirement to get informed consent was granted.

## Results

A total of 416 patients with CKD were included in our retrospective analysis. Around three-quarters of the patients were males (73%), and the mean age was 56 ± 15 years, as demonstrated in (Table 1). More than 50% of the patients were from the Middle East, followed by 36% from Asia. Around 77% of the patients lived with their families, while 21% lived with roommates.

Most patients had concurrent hypertension, stage V CKD, and diabetes mellitus (96%, 77%, and 64%, respectively). More than 75% of patients had CKD stage V and 20% had CKD stage IV. The vast majority of the patients (91%) had polypharmacy with more than five medications. Only 12% of the patients were fully vaccinated. In comparison, 73% received partial vaccination, as shown in (Table 2). More than half of the patients received pneumococcal vaccine and hepatitis B vaccine, 54% and 52%, respectively. In comparison, only 26% received the influenza vaccine as part of renal ambulatory care.

The factors evaluated to determine the predictors of vaccination included age, gender, Asian origin, employment, living conditions, concurrent medical conditions, CKD stage, allergy to medications, and use of injectable medications. Among the factors assessed, only stage V CKD was found to be a positive predictor of adhering to complete vaccination and partial vaccination in CKD (complete vaccination: OR 5.4 [95 confidence interval (CI) 1.6-18.7], p = 0.008; partial vaccination: OR 2.2 [95 CI 1.3-3.7], p=0.005) as demonstrated in (Table 3). Likewise, stage V CKD was a positive predictor of adhering to Influenza (OR 4.3 [95 CI 2-9.3], p < 0.001), Pneumococcal vaccine (OR 1.7 [95 CI 1-2.7], p = 0.044), and Hepatitis B vaccine (OR 2 [95 CI 1.2-3.3], p = 0.006). Another factor that increased the likelihood of receiving the influenza vaccine was diabetes mellitus (OR 2 [95 CI 1-4.1], p = 0.049). On the other hand, age older than 65 years (OR 0.5 [95 CI 0.3-0.8], p = 0.008) and confirmed allergy to oral medications (OR 0.5 [95 CI 0.2-1], p = 0.046) were found to be negative predictors of receiving Hepatitis B vaccine.

## Discussion

In our institution, all dialysis patients receive the complete recommended vaccines before starting the dialysis regimen. However, it remains unclear whether the non-dialysis CKD receives the most important, widely agreed upon vaccines for Hepatitis B, Influenza, and Pneumococcal.^16^ In this cross-sectional study, only 12% were fully vaccinated. In comparison, 73% received partial vaccination, and stage V CKD was a positive predictor of adhering to complete vaccination and partial vaccination in CKD. The gender disparity in our study is close to that in Qatar in general.^17^

The importance of these vaccines emerges from the fact that innate and adaptive immunological responses are affected by chronic kidney disease, so CKD patients are more susceptible to infections and decreased effectiveness of vaccines. The B and CD4_+_ T lymphocytes are declining, and the response to antigens is reduced. More importantly, there is an inadequacy in the production of antibodies after vaccination due to impairment in monocyte functions. Also, the phagocytosis capacity is decreased which results in impairment of neutrophil functions as well.^18–22^

Furthermore, impairment of the immune system can be attributed to additional factors such as uremia, mineral disorders, and inflammation that are present in almost all late CKD patients.^23^ Because of the different epidemiological priorities, there is no optimal global immunization policy for CKD patients, which results in varied vaccination regimes across countries.^10^

People with chronic illnesses generally have a lower tendency to receive the recommended vaccines than expected, even after ensuring their safety and huge beneficence.^24^ The Hepatitis B vaccine reduces the risk of infection by around 70 percent, and health status can be predicted by the patient’s response to vaccination in terms of seroconversion.^25,26^ The pneumococcal vaccine is highly recommended,^27^ given that Streptococcus pneumoniae is the most pneumonia-causing agent among CKD patients,^28^ and since it has a poor prognosis and higher mortality rate^27^. Annual Influenza vaccine is also recommended despite the limited evidence on its efficacy in CKD patients in particular.^29^

The prevalence of receiving the full recommended vaccines in our population under study is low, which might indicate that non-dialysis patients are not interested in getting these vaccines. The least received vaccine was the influenza vaccine, which could be because it is an annual vaccine. Reasons for non-adherence to vaccination include being afraid of side effects caused by vaccines, high cost in some settings, or simply that patients do not know about the need or availability of vaccines.^24^ A study showed that one reason for non-adherence toward Hepatitis B vaccination was the limited discussion between patients and their physicians regarding its benefits. Also, vaccination appointments should be of importance as well.^30^

The COVID-19 pandemic made the focus of CKD patients on receiving COVID-19 vaccines rather than other vaccines. Moreover, the COVID-19 pandemic forced many clinics to remain closed to the public for physical visits, and they were instead only providing telephone consultations, which may have affected the vaccine uptake rate. The COVID-19 situation has created many challenges to the health system in Qatar, especially in the hemodialysis department, where patients must have their sessions regularly.^31^ Many measures were taken to overcome these issues, such as designating a dialysis unit for COVID-19 patients, implementing a screening questionnaire to assess patients’ health status, and performing a rapid antigen test to identify COVID-19 cases.^32^

The presence of Diabetes Mellitus seems to encourage CKD patients to seek Influenza vaccination, as they receive more health educational materials, and they usually have more follow-up visits. Stage V CKD was the most potent positive predictor for all vaccines, and these patients may be more flexible in accepting vaccination compared to early stages of CKD, who may not recognize the importance of these vaccines.^33^ Also, in our institution, these patients are being followed up more frequently and are more likely to receive more health education materials in general.

We did not find any negative predictors for Pneumococcal and Influenza vaccines. Yet, the older ages and history of allergy to oral medications were associated with lower Hepatitis B vaccine uptake. It could be because older patients usually need more explanation and information regarding the vaccine’s safety and the possibility of not completing the three doses we considered not vaccinated. The 2-dose Hepatitis B vaccine could be a solution, and it has demonstrated high efficacy and cost-effectiveness^34^ and could promote better adherence.

A systematic review showed that to improve vaccination coverage, educating patients, tracking them, and offering health promotion sessions is essential.^35^ Patients of low educational/socioeconomic status should be provided more focus as studies showed generally lower vaccination rates among these groups.^36^ Unfortunately, given its secondary data nature, we did not examine this variable in our research.

Educational activities regarding the importance of vaccination have been proven effective during the COVID-19 era in Qatar,^37^ so it is one of the recommended interventions to improve vaccination rates among CKD patients.

Generally speaking, it is recommended that family physicians and specialists periodically check the vaccination status of their patients when encountered. Also, vaccination campaigns screen patients’ electronic records and phone patients who are not vaccinated to advise them on the process, benefits, and free vaccines.

This study was an observational study that has its limitations. First, due to the study’s retrospective nature, there was a risk of missing data. Second, the retrospective nature prevented acquiring information directly from the patients, which would have allowed us to investigate personal reasons for not taking the recommended vaccines and the opportunity to know whether patients had already taken the vaccine from other healthcare providers such as the private sector, or in their home country so it was not recorded in the system. Third, the study review period was during the COVID-19 era, which might have underestimated vaccination prevalence as attending ambulatory clinic appointments was not optimal due to COVID-19 safety measures. Nevertheless, in this study, we explored the rate of recommended vaccination among the vulnerable group of non-dialysis CKD patients, who might be overlooked compared to dialysis patients. Special vaccination attention is required for older patients and early-stage CKD patients as part of the mission to achieve The Global Vaccine Action Plan (GVAP), which focuses on the goal of life without vaccine-preventable illnesses for all communities.^38^

## Conclusions

Our study demonstrated low adherence to the recommended vaccines in non-dialysis CKD patients, with a prevalence of only 12%. Awareness of vaccination in CKD patients’ needs to be reemphasized to improve the adherence rates in Qatar, especially among early CKD stages who would benefit the most from the vaccination and are more likely to achieve seroconversion. Factors to be considered positively associated with higher vaccination rates were stage V CKD for all vaccines under study and Diabetes Mellitus for Influenza vaccine. We recommend initiating a public vaccine awareness campaign targeting CKD patients, plus raising this issue among family physicians who are the front liners and would encounter CKD patients in the early stages.

## Declaration of Competing Interests

The authors declare that they have no known competing financial interests or personal relationships that could have appeared to influence the work reported in this article.

## Data Availability Statement

The data supporting this study’s findings are available from the corresponding author upon reasonable request.

## Abbreviations

CKD: chronic kidney disease; ESRD: end-stage renal disease; AKI: acute kidney injury; GFR: glomerular filtration rate; DALY: disability-adjusted life years; GVAP: Global Vaccine Action plan.

## Funding

This research received no specific grant from funding agencies in the public, commercial, or not-for-profit sectors.

## Figures and Tables

**Table 1.** Baseline characteristics of patients with chronic kidney disease receiving renal ambulatory care (N = 416)

Characteristic	N (%)
Age (years)*	56 ± 15
**Gender**	
Male
Female	304 (73.1)
112 (26.9)
**Region of origin**	
Middle East	223 (53.6)
Asia	151 (36.3)
Africa	35 (8.4)
North America	3 (0.7)
Europe	2 (0.5)
South America	2 (0.5)
**Living conditions**	
Living with family	322 (77.4)
Living with roommates	89 (21.4)
Living alone	3 (0.7)
**Employment**	198 (47.6)
**Allergy**	
None	351 (84.4)
Allergy to oral medications	34 (8.2)
Allergy to injectable medications	19 (4.6)
Allergy to food	12 (2.9)
**Medical history**	
Chronic conditions other than CKD	413 (99.3)
Hypertension	399 (95.9)
Diabetes Mellitus	267 (64.2)
Heart diseases	136 (32.7)
Asthma	10 (2.4)
**CKD stage**	
1	4 (1.0)
2	3 (0.7)
3	5 (1.2)
4	84 (20.2)
5	320 (76.9)
**Number of chronic medications**	
<3	2 (0.5)
3-5	36 (8.7)
>5	378 (90.9)
**Injectable medication use**	302 (72.6)

* Reported as mean ± standard deviation; CKD: chronic kidney disease

**Table 2.** Prevalence of vaccination among patients with chronic kidney disease receiving renal ambulatory care (N = 416)

Vaccine	N (%)
Pneumococcal vaccine	226 (54.3)
Hepatitis B vaccine	215 (51.7)
Influenza vaccine	110 (26.4)
Partially vaccinated**	305 (73.3)
Fully vaccinated*	50 (12.0)

* Fully vaccinated is defined as receiving the three vaccines (influenza, pneumococcal, and hepatitis B);

** Partially vaccinated is defined as receiving one or two vaccines out of the three vaccines.

**Table 3.** Predictors of vaccination among patients with chronic kidney disease receiving renal ambulatory care (N = 416)

Characteristic	Full Vaccination		Partial Vaccination		Influenza Vaccine		Pneumococcal Vaccine		Hepatitis B Vaccine	
	Odds Ratio (95 CI)	P-value	Odds Ratio (95 CI)	P-value	Odds Ratio (95 CI)	P-value	Odds Ratio (95 CI)	P-value	Odds Ratio (95 CI)	P-value
Age > 65 years	0.5 (0.2-1.1)	0.074	0.8 (0.4-1.6)	0.494	1.6 (0.8-3.0)	0.147	1.3 (0.8-2.2)	0.402	**0.5 (0.3-0.8)**	**0.008**
Female gender	1.4 (0.7-3.0)	0.381	0.6 (0.3-1.1)	0.111	1.1 (0.6-1.9)	0.816	0.9 (0.5-1.4)	0.568	0.6 (0.4-1.0)	0.066
Asian origin	0.4 (0.1-1.2)	0.090	0.5 (0.3-1.1)	0.078	0.6 (0.2-1.3)	0.178	0.6 (0.4-1.2)	0.144	0.7 (0.4-1.3)	0.261
Employment	0.6 (0.3-1.4)	0.254	0.7 (0.4-1.3)	0.274	0.7 (0.4-1.4)	0.348	1.2 (0.7-2.0	0.558	0.7 (0.4-1.2)	0.206
Living with family	0.3 (0.02-3.4)	0.317	0.8 (0.1-8.1)	0.871	0.6 (0.1-6.3)	0.663	0.3 (0.03-2.8)	0.293	1.1 (0.2-7.0)	0.953
Living with roommates	0.1 (0.009-1.9)	0.135	0.4 (0.04-3.5)	0.379	0.1 (0.009-1.8)	0.123	0.2 (0.02-1.8)	0.142	0.7 (0.1-4.7)	0.742
Diabetes	2.4 (1.0-6.0)	0.059	1.2 (0.7-2.2)	0.470	**2.0 (1.0-4.1)**	**0.049**	1.2 (0.7-2.0)	0.468	1.2 (0.7-1.9)	0.575
Hypertension	2.8 (0.3-23.3)	0.342	0.9 (0.3-3.2)	0.919	0.5 (0.2-1.8)	0.320	0.8 (0.3-2.2)	0.621	1.6 (0.6-4.5)	0.375
Heart diseases	0.6 (0.3-1.2)	0.160	0.8 (0.4-1.4)	0.424	0.6 (0.4-1.1)	0.099	0.9 (0.6-1.5)	0.734	0.8 (0.5-1.3)	0.297
Stage V CKD	**5.4 (1.6-18.7)**	**0.008**	**2.2 (1.3-3.7)**	**0.005**	**4.3 (2.0-9.3)**	**<0.001**	**1.7 (1.0-2.7)**	**0.044**	**2.0 (1.2-3.3)**	**0.006**
Allergy to oral medications	2.1 (0.8-5.3)	0.135	0.5 (0.2-1.1)	0.079	1.6 (0.7-3.6)	0.256	1.0 (0.5-2.1)	0.962	**0.5 (0.2-1.0)**	**0.046**
Allergy to injectable medications	1.3 (0.4-4.6)	0.658	0.5 (0.2-1.4)	0.187	0.6 (0.2-2.0)	0.422	0.6 (0.2-1.5)	0.270	1.4 (0.5-3.8)	0.492
Injectable medication use	1.2 (0.5-3.1)	0.683	1.5 (0.9-2.6)	0.155	1.0 (0.5-2.0)	0.928	1.3 (0.8-2.2)	0.249	1.3 (0.8-2.2)	0.255
